# Immunocompetent Mouse Models in the Search for Effective Immunotherapy in Glioblastoma

**DOI:** 10.3390/cancers13010019

**Published:** 2020-12-23

**Authors:** Roxanne Wouters, Sien Bevers, Matteo Riva, Frederik De Smet, An Coosemans

**Affiliations:** 1 Laboratory of Tumor Immunology and Immunotherapy, Department of Oncology, Leuven Cancer Institute, KU Leuven, 3000 Leuven, Belgium; roxanne.wouters@kuleuven.be (R.W.); sien.bevers@kuleuven.be (S.B.); matteo.riva@kuleuven.be (M.R.); 2 Oncoinvent, A.S., 0484 Oslo, Norway; 3 The Laboratory for Precision Cancer Medicine, Translational Cell and Tissue Research Unit, Department of Imaging and Pathology, KU Leuven, 3000 Leuven, Belgium; frederik.desmet@kuleuven.be; 4 Department of Neurosurgery, Mont-Godinne Hospital, UCL Namur, 5530 Yvoir, Belgium

**Keywords:** glioblastoma, immunotherapy, model, animal model, preclinical, murine, immune response

## Abstract

**Simple Summary:**

Glioblastoma (GBM) remains the most aggressive brain tumor. Treatment typically includes surgery and radio/chemotherapy, but in spite of intensive treatment, virtually all tumors recur within the time-frame of months with insufficient and unsuccessful second line options. This clinical reality is in contrast to preclinical animal experiments, which often show successful outcomes of novel immunotherapeutic approaches. This discrepancy is largely explained by the small number of animal models and their limited capacity to mimic the complexity of the human disease. Moreover, new treatment options are typically administered as single treatments in animal models, whereas patients receive them in combination with standard-of-care. In this review, we provide an overview of the existing mouse models for GBM research and how each of them mimic (parts of) the human disease spectrum. As such we provide an overview of the advantages and limitations of the currently available options for in vivo drug testing for GBM.

**Abstract:**

Glioblastoma (GBM) is the most aggressive intrinsic brain tumor in adults. Despite maximal therapy consisting of surgery and radio/chemotherapy, GBM remains largely incurable with a median survival of less than 15 months. GBM has a strong immunosuppressive nature with a multitude of tumor and microenvironment (TME) derived factors that prohibit an effective immune response. To date, all clinical trials failed to provide lasting clinical efficacy, despite the relatively high success rates of preclinical studies to show effectivity of immunotherapy. Various factors may explain this discrepancy, including the inability of a single mouse model to fully recapitulate the complexity and heterogeneity of GBM. It is therefore critical to understand the features and limitations of each model, which should probably be combined to grab the full spectrum of the disease. In this review, we summarize the available knowledge concerning immune composition, stem cell characteristics and response to standard-of-care and immunotherapeutics for the most commonly available immunocompetent mouse models of GBM.

## 1. Introduction

Glioblastoma (GBM), is the most lethal brain tumor in adults, despite all therapeutic efforts [1,2]. After standard-of-care treatment, consisting of maximal surgical resection followed by radiotherapy (RT) and adjuvant temozolomide (TMZ), the median overall survival generally does not exceed 15 months [3,4,5]. This underscores the unmet medical need for the development of more efficient treatments. Several immunotherapeutic strategies, such as immune checkpoint inhibitors, cellular therapies and oncolytic viral therapies, have been explored in GBM [6,7]. However, to date all randomized clinical trials failed to provide lasting clinical efficacy [8,9,10,11,12], despite the many successes of pre-clinical studies [13,14,15]. We are therefore facing an important translational gap.

We believe that the discrepancy between preclinical and clinical results for immunotherapy in GBM can be explained by several factors, two of which play a pivotal role. First, current experimental models probably insufficiently mimic the complex situation in the human brain and are therefore unable to adequately predict the clinical scenario. In particular, the immune suppressive tumor microenvironment and its impact on immunotherapy has been mostly ignored or insufficiently characterized in previous preclinical studies [16,17]. Second, preclinical studies have rarely implemented the standard-of-care treatment (surgery, RT and TMZ) when testing the effect of immune modulators. This issue is particularly relevant for immunotherapy, since conventional treatments can modify the immune biology of GBM thereby altering the response to additional immunotherapy [8,18,19,20,21].

We believe that addressing these two problems would strongly boost the translational impact of GBM preclinical studies. However, integrating the full standard-of-care in preclinical research is challenging and require specific neurosurgical skills and equipment, which are not always available. Conversely, preclinical testing with multiple immunocompetent mouse models in order to better recapitulate multiple aspects of GBM biology and inter-patient heterogeneity is relatively straightforward. Nevertheless, to this end it is essential to know all relevant features of the available tumor models [22,23,24,25,26,27,28,29], in order to make an appropriate evaluation of which are the most adequate for each specific research question.

In this review, we will summarize the main features of the most relevant immunocompetent GBM mouse models (Table 1 and Table 2). For each model, we collected the available information on tumor immunity, cancer stemness, response to standard-of-care treatment and the effect of immunotherapeutics. The final goal will be to provide a useful tool for model selection and combination for the preclinical testing of new immunotherapeutic approaches against GBM.

## 2. Oldest Available Immunocompetent Mouse Models for GBM

**Figure 1:**
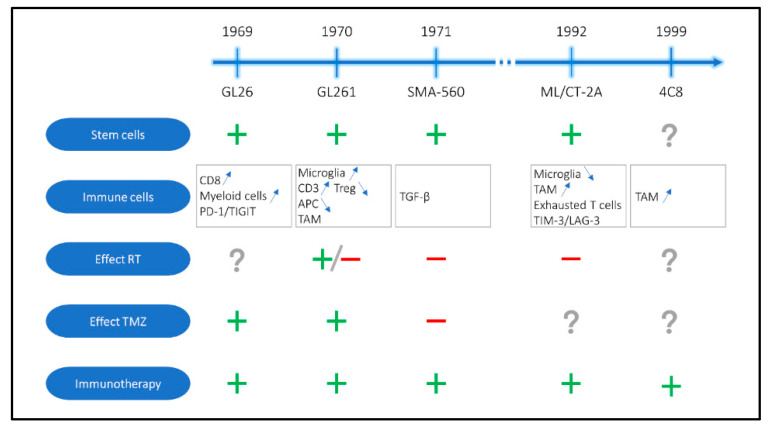
Schematic and chronological presentation of the old preclinical immunocompetent mouse models for glioblastoma with information about stemness, immune cell composition, the effects of standard of care and the efficiency of immunotherapy. RT: radiotherapy, TMZ: Temozolomide, PD-1: programmed cell death protein 1, TIGIT: T cell immunoglobulin and ITIM domain, Treg: regulatory T cell, TAM: tumor associated macrophage, TGF-β: transforming growth factor β, TIM-3: T cell immunoglobulin and mucin-domain containing 3, LAG-3: lymphocyte activation gene 3, (+) presence of stem cell populations and/or positive effect of treatment administration, (-) no effect of treatment administration and (?) data not available in literature.

The development and characterization of these oldest models has already been reviewed in detail in a previous publication by Oh et al. in 2014 [30]. Therefore, for these older models we will mainly focus on their most recent developments. An overview of these mouse models and relevant information can be found in Figure 1 and Table 1.

**Table 1:** Overview of the different characteristics of the GL26, GL261, ML/CT-2A, SMA-560 and 4C8 mouse models.

Model	Host	Induction	Histology	Immune Composition	Stem Cells	Effect of Standard-of-Care Therapy	Response to Immunotherapy	Reference
**GL261**	C57BL/6	Chemical induction with methylcholanthrene	GBM, ependymoblastoma	Immunogenic profile with high of frequency activated microglia and CD3^+^ T cells, low frequency of Tregs, presence of TAMs, low frequency of APCs	Stem cell like phenotype with Nestin and CD133 expression	RT: +/− TMZ: +	Survival benefit with several immunotherapeutic strategies in single and combination treatment (ICB, vaccination, virotherapy, …)	Ausman 1970 [15,21,31,32,33,34,35,36,37,38,39,40,41,42,43,44,45,46,47,48,49,50,51,52,53,54,55,56,57,58,59,60,61,62,63,64,65,66,67,68,69,70,71,72,73,74,75,76,77,78,79,80,81,82,83,84,85,86,87,88,89,90,91,92,93,94,95,96,97,98,99,100,101,102,103,104,105,106,107,108,109,110,111,112,113,114,115]
**GL26**	C57BL/6	Chemical induction with carcinogen implantation	GBM, ependymoblastoma	CD8^+^ T cell and myeloid cell infiltration with high expression of PD-1 and TIGIT immune checkpoints	Gene expression profile of glioma stem cells	TMZ: +	Generally positive	Sugiura 1969 [116,117,118,119,120,121,122,123,124,125,126,127,128,129,130]
**ML/CT-2A**	C57BL/6	Chemical induction with methylcholanthrene	Anaplastic astrocytoma	Overall immune suppressive microenvironment with low numbers of microglia, high numbers of resident macrophages and exhausted CD8^+^ T cells with TIM-3 and LAG-3 expression	Positive for CD133, Nestin and Oct4 stem cell markers	RT: -	Generally positive	Seyfried 1992 [26,33,77,131,132,133,134,135,136,137,138,139,140,141,142,143,144,145,146,147]
**SMA-560**	VM/Dk	Spontaneous	Anaplastic astrocytoma	Upregulation immunoregulatory pathways, TGF-β signaling	CD44 and Nestin expression when cultured in spheres	RT: - TMZ: -	Generally positive	Fraser 1971 [134,148,149,150,151,152,153,154,155,156,157,158,159,160]
**4C8**	B6D2F1	Clonal cell lines of a glial tumor from a transgenic mouse	Oligodendroglioma, astrocytoma	Large number of macrophages at the tumor periphery instead of in the tumor core	Not assessed	Not assessed	Generally positive (limited amount of data available)	Weiner 1999 [161,162,163,164,165]

GBM: glioblastoma, Treg: regulatory T cell, DC: dendritic cell, MHC-I/II: major histocompatibility complex I or II, TAM: tumor associated macrophage, APC: antigen presenting cell, PD-1: programmed cell death protein 1, TIGIT: T cell immunoglobulin and ITIM domain, TGF-β: transforming growth factor β, TIM-3: T cell immunoglobulin and mucin-domain containing 3, LAG-3: lymphocyte activation gene 3, RT: radiotherapy, TMZ: Temozolomide, ICB: immune checkpoint blockade. Bold: highlight.

### 2.1. GL261

#### 2.1.1. Origins and Tumor Characteristics

This chemically induced model was first developed in 1970 by Ausman et al. [116]. and has by far been the most widely used in glioblastoma research. In vivo, GL261 cells have been shown to express different general stem cell markers such as CD133 and nestin [31] while exhibiting infiltrative capacity of brain-tumor derived mesenchymal stem cells positive for Sox2, nestin, Sca-1, CD9, CD44 and CD166 [32]. Khalsa et al. performed a bulk RNA sequencing analysis on GL261 tumors which showed a strong enrichment of differentially expressed genes related to several immune pathways compared to naïve control mice, especially related to genes relevant for T cells, macrophages and eosinophils [33]. The same study also indicated a higher frequency of activated microglia, more total T cells, a lower frequency of regulatory T cells and antigen presenting cells compared to the ML/CT-2A tumor model [33]. All findings point towards the fact that the GL261 tumor model is more immunogenic than other models, such as the ML/CT-2A tumor model.

#### 2.1.2. Effect of Standard-of-Care

Both whole brain and focal beam irradiation strategies have been evaluated in the GL261 model. While whole brian irradiation was able to prolong survival and deliver long-term surviving mice, focal beam irradiation didn’t show the same potential [34,35,36,37]. Administration of TMZ was able to provide similar survival benefits in the GL261 model as is seen in GBM patients [38,39,40].

#### 2.1.3. Immunotherapeutic Approaches

Many different immunotherapies have been tested in the GL261 model. These include studies investigating programmed cell death protein 1 (PD-1) checkpoint blockade or other immune checkpoint inhibitors, oncolytic virotherapy, chimeric antigen receptor (CAR) T cell therapy, dendritic cell vaccination and many others [15,35,41,42,43,44,45,46,47,48,49,50,51,52,53,54,55,56,57,58,59,60,61,62,63,64,65,66,67,68,69,70,71,72,73,74,75,76,77,78,79,80,81,82,83,84]. In addition, the efficacy of many other less common immunotherapeutic approaches have been investigated in the GL261 models [85,86,87,88,89,90,91,92,93,94,95,96,97,98,99,100,101,102,103,104,105,106,107,108,109]. The vast majority of these therapies showed promising results, with a stronger anti-tumor response and improved survival rates. These immunotherapeutic strategies have been investigated as single treatments, in combination with other types of immunotherapies or in combination with the standard-of-care treatment. However, only part of the standard-of-care (usually TMZ, less commonly RT or RT-TMZ) was taken into consideration [39,40,78,79,110,111,112,113,114]. Interestingly, the efficacy of checkpoint inhibition directed against PD-1 or its ligand (PD-L1) in combination with TMZ, RT, or both was tested in six, two or one preclinical studies, respectively. Out of these eight combinatorial studies, seven were conducted with the GL261 tumor model [21].

The effects of steroids, largely used in the clinic to reduce brain oedema in GBM patients, were analyzed in one study using the GL261 tumor model. This study demonstrates that steroids have an inhibitory effect on anti-tumor immunity and that blocking cytotoxic T-lymphocyte-associated protein 4 (CTLA-4), but not PD-1, could partially prevent such negative modulation [115].

### 2.2. GL26

#### 2.2.1. Origins and Tumor Characteristics

The GL26 model is the oldest immunocompetent preclinical model for GBM and has been developed in 1969 by chemical induction [116]. It has been less extensively used than the (similar) GL261 model. Although both models show a great histological resemblance, the main difference is that GL26 tumors show a large extent of necrosis and vascularity and therefore tend to be more hemorrhagic [116]. Crommentuijn et al. [117] described the presence of a tumor antigen-specific CD8^+^ T cell population which displays a tolerogenic phenotype with a high expression of several immune checkpoints such as PD-1 and T cell immunoglobulin and ITIM domain (TIGIT). The infiltration of myeloid cells expressing these immune checkpoint ligands was also observed [117]. Furthermore, the importance of galactokinase (Gal1) in the immune suppression of the GL26 model was described, since it masks tumor cells from immune recognition [118,119]. The importance of glial toll-like receptor 2 (TLR2) as a bridge between the innate and the adaptive immune response was also reported, which is crucial in providing an effective immune response against the tumor [120]. Genetic analysis of GL26 tumors also revealed a specific acquisition of several stem cell markers that were correlated to anti-tumor T cell activity [121].

#### 2.2.2. Effect of Standard-of-Care

Radiotherapy (also if as whole body irradiation) and TMZ as a monotherapies have both been proven to be effective in prolonging survival in the GL26 mouse model [122,123,124,125,126]. Furthermore, TMZ treatment was able to increase cross-priming of tumor antigen-specific CD4^+^ T cells and CD8^+^ T cells and suppressed the frequency of regulatory T cells (Tregs) [125].

#### 2.2.3. Immunotherapeutic Approaches

GBM is strongly invasive and tumor cells can be found embedded in the normal parenchyma at great distance from the main tumor. This makes a complete resection not feasible [117]. Yadav et al. analyzed this problem with the GL26 model, and they found that down regulation of C-X-C chemokine receptor type 4 (CXCR4) led to less perivascular invasion and increased survival. Furthermore, CXCR4 knockdown sensitizes the tumors to irradiation, making this molecule an interesting therapeutic target [127]. Another novel therapeutic strategy targets the proton/H^+^ eflux mechanism important for the maintance of the intracellular pH. The inhibition of the H+ eflux mechanism (NHE1) reduced tumor volume, invasion and prolonged overall survival in GL26 (and SB28) glioma models. This type of treatment resulted in an accumulation of CD8^+^ T cells and sensitized animals to anti-PD-1 therapy [128]. Also the mTOR pathway is a frequent target of anti-glioma therapy. Targeting this pathway with rapamycin in combination with immunotherapy had a synergistic effect and a long term survival advantage. Rapamycin administration also resulted in a long lasting central memory CD8^+^ T cell response and a stronger anti-tumor response after a second tumor challenge [129]. The combination of TMZ with interferon (IFN)-β was also tested in the GL26 model and showed enhanced anti-tumor effects compared to TMZ alone [130].

### 2.3. ML/CT-2A

#### 2.3.1. Origins and Tumor Characteristics

The CT-2A model was first described by Seyfried et al. in 1992 [131] and accurately represents numerous GBM characteristics, including the intra-tumoral cellular heterogeneity and the proliferative and metabolic profiles [132]. CT-2A cells cultured as monolayer cells (ML/CT-2A) in vitro express different stem cell markers such as CD133, nestin and Oct4 [26]. Also in vivo the expression of CD133 and Nestin is observed in ML/CT-2A tumors [133], indicating that the cells keep their stemness during tumor growth in mice. Khalsa et al. performed RNA sequencing to identify the ML/CT-2A immune profile in vitro [33]. In contrast to the highly immunogenic GL261 model, ML/CT-2A cells showed no enrichment of any immune response-related pathway. In vivo, ML/CT-2A tumors had lower numbers of CD45^low^CD11b^low^CX3CR1^+^ microglial cells (considered activated or resting based on MHCII positive or negative staining, respectively), but higher numbers of CD11b^+^F4/80^+^CD64^+^Ly6C^−^ resident macrophages and CD39^+^Tim3^+^Lag3^+^CD8^+^ exhausted cytotoxic T cells compared to other glioblastoma tumor models. Furthermore, 70–80% of T cells in the tumor microenvironment of ML/CT-2A tumors exhibit prolonged expression of T cell immunoglobulin and mucin domain-containing protein 3 (TIM-3) and lymphocyte-activation gene 3 (LAG-3), both markers for dysfunctional T cells [33]. Overall, the ML/CT-2A model is characterized by an immune suppressive tumor microenvironment and exhausted effector T cell function, making it a very suitable tumor model for GBM research in the field of immunotherapy since a similar immune phenotype is observed in GBM patients [134].

#### 2.3.2. Effect of Standard-of-Care

The ML/CT-2A model has not been widely used in preclinical studies assessing the effects of the standard-of-care treatment. Only one study described the effects of RT in the model [135], where whole brain irradiation was ineffective in prolonging survival in mice. As already mentioned, the surgical removal has an influence on the immune composition of the remaining/recurrent tumor, with obvious implications for immunotherapies. Nevertheless, this treatment has only occasionally been assessed in preclinical studies. Khalsa et al. performed an immunophenotyping of the ML/CT-2A mouse model before and after surgical resection of the tumor [33]. After tumor resection, an increase of CD4^+^ and CD8^+^ T cells and activated microglia was observed with a decrease of resting macrophages and resident microglia. Furthermore, PD-1 expression decreased and CD25 expression increased on CD4^+^ T cells post-tumor resection [33]. These data suggest that tumor resection in the ML/CT-2A model partially removes the immune suppressive microenvironment and promotes immune activation, possibly creating a favorable momentum for administration of immunotherapies.

#### 2.3.3. Immunotherapeutic Approaches

Given the disappointing results of checkpoint blockade in GBM patients, the current focus of preclinical research in this field is on combining checkpoint blockade with newly identified targets such as interleukin 6 (IL-6), IL-7, IL-12 or phagocytosis pathways, in order to overcome T cell exhaustion [136,137,138]. In the ML/CT-2A tumor model, the combination of PD-1 checkpoint inhibition with anti-CD137 decreased TIL exhaustion, improved TIL functionality and resulted in 50% long term survivors [139]. Another novel combination treatment recently tested was the combination of anti-PD-L1 with gene-mediated cytotoxic immunotherapy which resulted in more long term survivors as compared to the monotherapies [140]. One last focus of interest has been to improve the delivery of checkpoint inhibitors via lipid nanoparticles. In combination with RT, this strategy led to a depletion of tumor-associated myeloid cells and a significantly improved survival in ML/CT-2A and GL261 models [135].

Oncolytic virus (OV) therapy has great potential for success, however the best balance between maximal anti-tumor activity and acceptable toxicity is difficult to find, especially following direct intracranial infusion. Certain OVs based on herpes simplex virus (HSV) are safe but have only little anti-tumor response. To overcome this limitation Passaro et al. engineered an HSV to express an antibody against PD-1 and injected it intratumorally. This resulted in an increased median survival and immune memory against the both ML/CT-2A and GL261 tumors [141]. The combination of OV therapy with PD-1/PD-L1 immunotherapy provided a synergistic effect leading to an improved overall survival and an activation of the immune response capable to reverse the tumor-induced immune suppression [142,143]. On the other hand, OVs based on vesicular stomatitis virus (VSV) have a very robust anti-tumor effect but are extremely neurotoxic when injected in the brain. Therefore, Balathasan et al. [144] used an intravenous pretreatment of VSVΔ51 as a way to induce peripheral immunization before intracranial injection of an otherwise lethal dose of VSVΔ51. This resulted in complete tumor regression in 20% of ML/CT-2A tumor bearing mice. Also OVs based on Semliki Forest Virus (SFV) have been developed [145,146]. When injected intravenously, they resulted in a prolonged survival with 27% of the mice bearing ML/CT-2A tumors cured, whereas there was no significant effect in the GL261 model [146].

In an interesting study, Ladomersky et al. used the ML/CT-2A model to demonstrate increased immune suppression, decreased immunotherapeutic efficacy and decreased survival in old age animals (75 week old mice, corresponding to 58–59 year old humans) [77]. The impact of age on GBM development and treatment has been ignored most of times: in the majority of studies, animals of young age (6–12 weeks, corresponding to early adulthood in humans) are used for preclinical GBM research [147]. Given that the median age at diagnosis for GBM is 65 years, the age difference between tumor models and patients is extremely relevant.

### 2.4. SMA-560

#### 2.4.1. Origins and Tumor Characteristics

The SMA-560 model is one of the few models that spontaneously arose in VM/Dk mice as initially described by Fraser et al. in 1971 [148]. It was established as a cell line in 1980 by Serano and colleagues [149]. The fact that the model developed spontaneously in immunocompetent mice, makes it a very interesting model to study. A genetic characterization of the model revealed an upregulation of genes involved in antigen presentation, interferon-related protein expression and a general increase in genes related to immunoregulatory pathways indicating the presence of an ineffective immune response in the tumor microenvironment of the SMA-560 model [150]. Furthermore, the immune suppressive protein transforming growth factor beta (TGF-β) has been shown to play an important role in SMA-560 tumor development [151]. The expression of PD-1, TIM-3 and LAG-3 on tumor infiltrating lymphocytes is also increased in SMA-560 tumors [134]. In terms of stemness characteristics, it has been described that in vitro SMA-560 cells express only a limited amount of CD44 and nestin stem cell markers. However, when cultured in sphere cultures the cells seem to increase their CD44 and Nestin expression, which was correlated with a more aggressive tumor behaviour in vivo [152]. Schneider and colleagues described the difference in tumorigeneic potential in young and old VM/Dk mice. Interestingly at baseline, older SMA-560 mice had a significantly worse survival as compared to younger mice, in contrast to the GL261 model where this difference was not observed [153].

#### 2.4.2. Effect of Standard-of-Care

In vitro, SMA-560 cells were highly resistant to TMZ treatment and only responded to high doses of irradiation [152]. However, only a few studies assessed these effects in vivo [154]. Whole brain irradiation as a single treatment or combined with TMZ was either ineffective or provided only a limited and non-significant improvement in survival compared to control mice [154,155]. This indicates that the standard-of-care used in GBM patients is ineffective in prolonging survival of the SMA-560 mouse model. Therefore, the translational potential of the model should be considered carefully when translating results to the whole GBM patient population. However, a tumor model that does not respond to RT or TMZ can be relevant in studying treatment options for patients who respond poorly to this standard-of-care treatment regimen or for the recurrent situation where resistance appeared.

#### 2.4.3. Immunotherapeutic Approaches

An important immunological therapeutic target studied in the SMA-560 model is excessive TGF-β signaling [156,157]. As such, the administration of phosphorothioate-locked nucleic acid (LNA)-modified antisense oligonucleotide gapmers targetting TGF-β resulted in prolonged survival and increased CD3^+^ and CD8^+^ cytotoxic T cell infiltration [158]. Another emerging treatment strategy that has been tested in this model is CAR T cell therapy, which was shown to generate a pro-inflammatory tumor microenvironment and to significantly extend survival in the SMA-560 model [159]. Furthermore, anti-angiogenic treatment has a positive effect on survival in the SMA-560 model [155]. One of the problems in GBM treatment is the delivery of the compound trough the blood brain barrier. In this regard, microbubbles have been tested to increase the local concentration of certain types of treatments. This strategy was succesfully tested for doxorubicin in the SMA-560 model [160].

### 2.5. C8

#### 2.5.1. Origins and Tumor Characteristics

The 4C8 model was established in 1999 from clonal cell lines of a glial tumor (MOCH-1) in B6D2F1 mice [161]. Gazdzinski et al. [162] compared the characteristics of this model with the GL261 model. The 4C8 model is less aggressive, the tumor has higher cell density, less necrosis and invasiveness with a more normal vasculature and less mitotic cells as compared to the GL261 model. Both models have a large number of infiltrating macrophages; however, these cells are located at the tumor periphery in the 4C8 model [162]. All these features, consistently pointing towards a lower aggressiveness in comparison to the GL261 model, have strongly limited the used of the 4C8 tumor model.

#### 2.5.2. Immunotherapeutic Approaches

This model has been used very limitedly in immunotherapeutic or anti-angiogenic research [163,164]. The combination of an anti-angiogenic receptor tyrosine kinase inhibitor with a proteasome inhibitor resulted in a significantly improved survival and an induction of anti-angiogenic effects which leads to vascular normalization [164]. The effects of oncolytic virotherapy were assessed in the model as well. In vitro, 4C8 cells showed the same sensitivity as human glioma cells to a series of type HSV-1 [165]. In vivo studies showed a prolongation of survival with an intracranial injection of an IL-12-expressing HSV [165].

## 3. Recently Developed Immunocompetent Mouse Models for GBM

**Figure 2:**
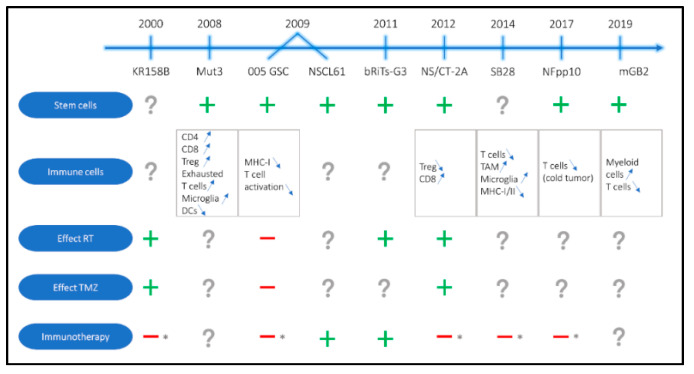
Schematic and chronological presentation of the more recent preclinical immunocompetent mouse models for glioblastoma with information about stemness, immune cell composition, the effects of standard of care and the efficiency of immunotherapy. RT: radiotherapy, TMZ: Temozolomide, Treg: regulatory T cell, DC: dendritic cell, MHC-I/II: major histocompatibility complex I or II, TAM: tumor associated macrophage, (+) presence of stem cell populations and/or positive effect of treatment administration, (−) no effect of treatment administration, (?) data not available in literature and (*) resistance to immune checkpoint blockade.

Various new GBM models have been developed in the last years. In most cases, this has been done by means of viral vectors which were either used to manipulate isolated mouse cells (mGB2, NSCL61 and bTiTs-G3) or injected directly into the animals’ brain (SB28, 005 GSC and NFpp10 models). Additionally, tumor models have been generated from spontaneously developed tumors in genetically altered mice (KR158B and Mut3) or by culturing older cell lines in a different way (CT-2A). In all cases, stable cells lines amenable of standard intracranial injection have been obtained [22,23,24,25,27,28,166]. An overview of these mouse models and relevant information can be found in Figure 2 and Table 2.

**Table 2:** Overview of the different characteristics of the KR158B, Mut3, 005 GSCs, NSCL61, bRiTs-G3, NFpp10-GBM, NS/CT-2A, SB28 and mGB2 mouse models.

Model	Host	Induction	Histology	Immune Composition	Stem Cells	Effect of Standard-of-Care Therapy	Response to Immunotherapy	Reference
**KR158B**	C57BL/6	Spontaneous tumor development in Nf1 and p53 mutant mice	Secondary GBM	Not assessed	Not assessed	RT/TMZ: +	Resistance to ICB	Reilly 2000 [12,29,65,166,167,168]
**Mut3**	C57BL/6	Spontaneous tumor development in Nf1, p53 and Pten mutant mice	GBM, high-grade astrocytoma	High levels of classical and exhausted CD8^+^ T cells, CD4^+^ T cells, Tregs and resting microglia and low levels of DC infiltration	Increased GFAP and Nestin expression	Not assessed	Not assessed	Kwon 2008 [33,169,170]
**005 GSCs**	C57BL/6	Transduction in hippocampus of adult mice with vectors with activated HRas en AKT	GBM, heterogeneous	Relatively non-immunogenic, absence of MHC-I and down regulation of co-stimulatory molecules, limited T cell activation, strong correlation with human tumor immune microenvironment	Glioma stem cell tumor model	Not assessed	Resistance to ICB	Marumoto 2008 [24,33,168,171,172,173,174,175]
**NSCL61**	BALB/c	HrasL61 overexpression in p53 deficient neural stem cells	GBM, heterogeneous	Not assessed	Tumor model is derived from neural stem cells	Not assessed	Generally positive (limited amount of data available)	Hide 2009 [27,68,72]
**bRiTs-G3**	C57BL/6	Overexpression of HRasV12 in neural stem cells from mice with homozygous deletion of the Ink4a/Arf locus	GBM, mesenchymal	Not assessed	Tumor model is derived from neural stem cells	RT: + RT resistance develops after repeated exposure	Generally positive (limited amount of data available)	Sampetrean 2011 [28,68,176]
**NFpp10-GBM**	C57BL/6	Embryonic stem cells infected with shp53-shNf1 and shPten lentiviral vector	GBM	Lack of T cell infiltration	Tumor model is derived from neural stem cells	Not assessed	Resistance to ICB	Allen 2017 [13,24,25,177]
**NS/CT-2A**	C57BL/6	Culturing of CT-2A cells in serum-free stem cell culture medium	Astrocytoma	Decrease in number of Tregs and increased CD8^+^ T cells compared to ML/CT-2A	Increased expression of Nestin and CD133 expression compared to ML/CT-2A	RT: + TMZ: + RT/TMZ: +	Resistance to ICB	Binello 2012 [21,26,30,133,178]
**SB28**	C57BL/6	Intraventricular transfection of Nras, PDGF and shp53 in neonates	GBM, proneural	Weakly immunogenic: few infiltrating T cells, abundant macrophage and microglial infiltration, absence of MHC-I and MHC-II expression	Not assessed	Not assessed	Resistance to ICB	Kosaka 2014 [9,12,23,58]
**mGB2**	C57BL/6	p53 and Pten deficient neural stem cells in adult mice	GBM, mesenchymal	Strong presence of myeloid cells and only few lymphocytes	Tumor model is derived from neural stem cells	Not assessed	Not assessed	Costa 2019 [22,179]

GBM: glioblastoma, Treg: regulatory T cell, DC: dendritic cell, MHC-I/II: major histocompatibility complex I or II, RT: radiotherapy, TMZ: Temozolomide, ICB: immune checkpoint blockade, GFAP: glial fibrillary acidic protein, PDGF: platelet-derived growth factor, Pten: phosphatase and tensin homolog, Nf1: neurofibromin 1, Nras: neuroblastoma reticular activating system, Hras: Harvey rat sarcoma viral oncogene homolog. Bold: highlight.

### 3.1. KR158B

#### 3.1.1. Origins and Tumor Characteristics

This mouse model was developed in 2000 by Reilly et al. and it is the first astrocytoma mouse model that was generated by knocking-down neurofibromin 1 (Nf1) and tumor protein p53 in mice which then spontaneously developed brain tumors with variable histology from low grade astrocytoma to GBM [29]. KR158B is the cell line derived from the most aggressive variants, which recapitulate the main features of human GBM [166]. To date, information on the immune and stemness characterization of this model is not available yet.

#### 3.1.2. Effect of Standard-of-Care

The administration of whole brain irradiation and TMZ as single treatments wasn’t able to positively affect survival in the KR158B model, and the combination of both resulted in a small median survival benefit of only five days, in line with results in the most aggressive human GBMs and making this a promising model for future preclinical research [166].

#### 3.1.3. Immunotherapeutic Approaches

In the KR158B model, the combination of myeloablative conditioning, dendritic cell (DC) vaccination and adoptive cellular therapy resulted in a doubeling of the median survival and 30% of cured mice [166]. This model has also been used to test alternative TMZ treatment schedules in combination with immunotherapy [65,167,168]. The combination of TMZ and anti-PD-1 treatment has been shown to decrease the expression of T cell exhaustion markers. However, this had no effect on survival indicating that the model can develop resistance mechanisms to both these treatments [65]. However, the combined inhibition of PD-1 and C-C chemokine receptor type 2 (CCR2) lead to a synergistic effect and improved mouse survival, overvcoming the resistance to anti-PD-1 monotherapy [168]. The recent failure of clinical trials involving anti-PD-1 treatment [12] has demonstrated that human GBM are able to promote strong resistance mechanisms hampering the efficacy of checkpoint inhibitors. Therefore, performing preclinical research in models showing the same type of resistance, such as the KR158B, is of the utmost importance for an appropriate design of future clinical trials.

### 3.2. Mut3

The Mut3 tumor model was developed by Kwon et al. [169] in 2008 by generating *Nf1*, *p53* and *Pten* deficient mice which subsequently developed spontaneous high-grade astrocytomas. Neural stem cells (NSCs) from presymptomatic mice already showed aberrant stem cell features including higher proliferation levels, increased glial fibrillary acidic protein (GFAP) and increased Nestin expression [170]. The Mut3 cell line was generated by isolating the spontaneously developed tumors and bringing them in culture where they are maintained in neurosphere conditions [169]. Mut3 tumors are immunologically characterized by high levels of both classical and exhausted infiltrating CD8^+^ T cells, CD4^+^ T cells, Tregs, and resting microglia, and by low levels of DC infiltration [33]. At this moment, no data is available on effects of standard-of-care or immunotherapeutics in the model.

### 3.3. 005 GSCs

#### 3.3.1. Origins and Tumor Characteristics

Marumoto et al. [24] developed this mouse model by injecting Cre-*loxP*–controlled lentiviral vectors expressing activated oncogenes AKT and Harvey-Ras in the hippocampus of GFAP-Cre Tp53+/− mice. Subsequently, the obtained tumor cells were cultured as neurospheres and the 005 GSCs cell line was established [24,171]. Next, Saha et al. developed an immunocompetent model by reinjecting the 005 GSC cells in C57BL/6 mice [172,173]. 005 GSC-derived tumors show the same features as the primary tumor. Furthermore, 005 GSC cells express several stem cell markers such as Nestin, CD133 and Sox2 and proangiogenic vascular endothelial growth factor (VEGF) both in vitro and in vivo [173]. Even though RNA seq analysis performed by Khalsa et al. [33] showed that 005 GSC tumors exhibit a more immunologically active profile, Cheema et al. [173] described the tumors as non-immunogenic with the absence of major histocompatibility complex (MHC)-I expression and down regulation of co-stimulatory molecules. Nonetheless, Khalsa et al. showed that 005 GSC tumors had large amounts of activated and resting microglia and CD4^+^ Tregs, but low numbers of classical and exhausted CD8^+^ T cells [33]. This immunological phenotype strongly correlates with the immune microenvironment of GBM tumors in patients, making it a highly translational mouse model to be used for preclinical studies involving immunotherapeutic GBM research [33].

#### 3.3.2. Effect of Standard-of-Care

Saha et al. [174] demonstrated that both low and high doses of TMZ treatment were ineffective in providing a survival benefit in the 005 GSC tumor model. In combination with OV, TMZ even counteracted the OVs positive effect on survival, indicating the chemo-resistant nature of the 005 GSC tumor model and the importance of implementing standard-of-care treatment in preclinical research. The effects of RT on 005 GSC tumors have not yet been described.

#### 3.3.3. Immunotherapeutic Approaches

005 GSC model has experienced occasional use in immunotherapy research to evaluate the effect of combination treatments with OV, VEGF receptor (VEGFR) tyrosine kinase inhibitors (TKI) and immune checkpoint blockade [172,173,175]. Cheema et al. [173] showed the effect of a genetically engineered oncolytic HSV armed with IL-12 (G47Δ-mIL12). Median survival was prolonged after intratumoral injection of G47Δ-mIL12. Treatment with G47Δ-mIL12 doesn’t only target GSCs but also increases IFN-γ release, inhibits angiogenesis, and reduces the number of Tregs in the tumor [173]. The combination of G47Δ-mIL12 with the VEGFR TKI axitinib, anti-CTLA-4, anti-PD-1 or anti-PD-L1 further enhanced the positive effects on survival [172,175] while monotherapy of checkpoint inhibition with anti-CTLA-4, anti-PD-1 or anti-PD-L1 only showed positive but modest effects [172]. Interestingly, a triple combination of G47Δ-mIL12 with anti-CTLA-4 and anti-PD-1 showed a synergistic curative effect that was accompanied with M1 macrophage polarization and an increased CD8^+^ T cell / Treg ratio [172]. Additionally, targeting myeloid-derived suppressor cells (MDSCs) by using a CCR2 antagonist was able to sensitize 005 GSC tumors to anti-PD-1 therapy [168].

### 3.4. NSCL61

#### 3.4.1. Origins and Tumor Characteristics

The NSCL61 model was originally developed by Hide et al. [27] in 2009 by the overexpression of oncogenic Harvey rat sarcoma viral oncogene homolog (HRas)^L61^ in p53 deficient NSCs that subsequently formed tumors in nude mice after stereotactic injection. These tumors were grown in culture as the NSCL61 cells and consist of an heterogenous population of both glioma initiating and non-tumorigenic cells [27]. An immunocompetent tumor model can be established by injecting NSCL61 cells stereotactically in C57BL/6 mice [68]. An immunological evaluation of NSCL61 tumors has not yet been performed.

#### 3.4.2. Immunotherapeutic Approaches

The NSCL61 has only been sparsly used in preclinical GBM research [68,72]. Tumor cell lysate-based vaccination therapy in combination with immunotherapy targeting CD40 resulted in the induction of IFN-γ secretion from CD4^+^ T cells and prolonged survival [72]. The local delivery of anti-CD40 monoclonal antibodies resulted in an increased apoptosis, T cell infiltration and significantly prolonged survival in the NSCL61 and bRiTs-G3 model, but not in the GL261 model due to a lower CD40 expression [68].

### 3.5. bRiTs-G3

#### 3.5.1. Origins and Tumor Characteristics

Sampetrean et al. [28] developed the bRiTs-G3 model in 2011 by retroviral transduction of constitutively active HRas^V12^ in normal neural stem/progenitor cells isolated from the subventricular zone of adult mice with a homozygous deletion of the *Ink4a/Arf* locus. Brain tumor-initiating cells were subsequently cultured as neurospheres. Molecular characterization of bRiTs-G3 tumors showed expression of mesenchymal and stem cell markers indicating a mesenchymal GBM subtype [28].

#### 3.5.2. Effect of Standard-of-Care

The bRiTs-G3 tumor model was used to study resistance to RT by exposing the cells in vitro to repeated cycles of irradiation. After stereotactic injection of the pretreated cells, bRiTs-G3 tumors were resistant to subsequent treatment with RT, indicating the bRiTs-G3 cells acquire a radio-resistant phenotype after repeated exposure to irradiation [176].

#### 3.5.3. Immunotherapeutic Approaches

When the bRiTs-G3 cells acquire their radioresistant phenotype, this also results in upregulation of insulin-like growth factor 1 receptor (IGF1R). Therefore, IGF1R blockade has been proposed as treatment option to prevent RT resistance and recurrence after RT [176]. Additionally, the bRiTs-G3 models has been used in immunotherapy research with anti-CD40 treatment where it significantly prolonged survival compared to control mice [68].

### 3.6. NFpp10-GBM

#### 3.6.1. Origins and Tumor Characteristics

NFpp10-GBM cells were created in 2017 by infecting embryonic C57Bl/6 NSCs with lentiviral vectors containing shP53-shNF1 and shPten [13,24,25]. To date, this model has not yet been fully characterized and has only experienced very limited use in preclinical GBM research.

#### 3.6.2. Immunotherapeutic Approaches

The NFpp10-GBM model is mainly used to study tumor vasculature and angiogenesis [13,177]. The combination treatment of VEGF inhibition and anti-PD-L1 had no significant effect on survival. The ineffectiveness of the combination treatment was not due to the lack of PD-L1 expression of the cells, but rather the lack of T cell infiltration into the tumor [13]. To increase treatment efficacy a vascular targeting peptide (VTP) was developed containing the tumor necrosis factor (TNF) superfamily cytokine LIGHT which stimulates T cells, promotes vascular inflammation and is involved in lymph node neogenesis. Triple treatment with LIGHT-VTP, anti-VEGF and anti-PD-L1 resulted in a significantly reduced tumor burden as compared to untreated controls. Additionally, this combination treatment amplified high endothelial venules’ frequency and T cell accumulation [177].

### 3.7. NS/CT-2A

#### 3.7.1. Origins and Tumor Characteristics

As highlighted in the already mentioned review by Oh et al. [30], culturing CT-2A cells in neurospheres (NS/CT-2A) results in an increase of their stemness features. However, the difference in immunogenicity between CT-2A cells cultured in ML and NS was not described yet [26]. In a study performed by our group in 2019, NS/CT-2A tumors have been shown to induce a shorter survival and a higher expression of stemness and vascular markers compared with their ML counterpart. Furthermore, NS/CT-2A tumors showed an increase in CD8^+^ T cells and a decrease in the number of Tregs compared to ML/CT-2A tumors [133]. These features of the NS/CT-2A tumor model make it suitable for preclinical research aimed at developing therapeutic strategies against tumor stem cells and immune suppression.

#### 3.7.2. Effect of Standard-of-Care

In the NS/CT-2A model, TMZ and stereotactic RT were able to prolong survival when administered as monotherapies or in combination. As monotherapy, stereotactic RT positively modulated both the adaptive and the innate immune system (increased CD8^+^ T cells and decreased M2 macrophages and monocytic MDSCs (mMDSCs)) while TMZ only improved innate immunity (reduced mMDSCs) and to a lower extent than stereotactic RT [21]. Interestingly, the combination of these two treatments, despite prolonging survival, was immunologically detrimental compared to RT alone. This model was also used to assess the effects of stereotactic RT dose-escalation and dose-fractionation. RT dose-escalation was associated with prolonged survival, improved anti-tumor immunity and reduced expression of stem cell markers. Conversely, RT dose-fractionation drastically reduced this positive effect [178]. Given the fact that GBM patients are currently treated with a fractionated RT schedule, these results highlight the need for studies aimed at identifying new RT schedules capable to induce a better immune modulation and a more efficient combination with immunotherapeutics.

#### 3.7.3. Immunotherapeutic Approaches

As already mentioned, the combination of stereotactic RT and TMZ in the NS/CT-2A model induced a less favorable immune microenvironment compared to RT alone. The model also appeared quite resistant to anti-PD-1 since this treatment could only induce minor modifications of survival and tumor immunity when administered alone or following RT-TMZ [21].

### 3.8. SB28

#### 3.8.1. Origins and Tumor Characteristics

The SB28 cell line was developed by Kosaka et al. [23] via intraventricular injection of the oncogenes neuroblastoma reticular activating system (NRas), platelet-derived growth factor (PDGF) and short hairpin p53 in neonate C56BL/6 mice. Seven weeks following glioma induction, brain tissue was harvested, minced and seeded. The clone with the highest luciferase activity was selected and the SB28 cell-line was established [23]. There was an inverse correlation between the number of injected SB28 cells and the median survival [58]. The tumors can be classified as proneural, as indicated by the presence of PDGF alterations, and they are weakly immunogenic, as is the case for human GBMs [23,58]. High cellularity of the tumor area, invasion of the normal brain parenchyma and areas of hypervascularization are also common characteristics of SB28 tumors and human GBM. Very few infiltrating T cells can be found, in contrast to abundant macrophage and microglial infiltration. Due to the absence of constitutive MHC-I and MHC-II expression, SB28 tumors are less susceptible of T cell immunosurveillance compared to GL261 tumors. SB28 tumors exhibit a very low mutational load (50-fold less than GL261 tumors), resulting in only a few neoepitopes and explaining the weak immunogenicity. The mutated genes were equally distributed across several pathways, but 10% of all mutations were found in the PDGF signaling pathway, confirming the proneural classification [58].

#### 3.8.2. Immunotherapeutic Approaches

The use of combined anti-PD-1 and anti-CTLA-4 was curative in over 50% of GL261 bearing mice, whereas it was ineffective in SB28 tumors [58]. This indicates that the SB28 model is more representative to human disease where immune checkpoint blockade provided unsatisfactory results so far [9,12]. Another study investigated the modulation of CD40 signaling and cyclooxygenase (COX)-2 blockade in the SB28 and GL261 models. The combination strategy promoted M1 cells, enhanced T cell effectors and prolonged survival [23].

### 3.9. mGB2

The mGB2 tumor model was generated by Costa et al. [22] in 2019 by means of a double knockout (DKO) of *Pten* and *p53* specifically in NSCs. Histopathological analysis of the developed tumors showed microvascular proliferation, necrotic areas and positivity for markers such as GFAP, oligodendrocyte transcription factor (OLIG2) and Ki67, all characteristics of human high-grade gliomas [22]. Subsequently, NSCs were isolated from the DKO mice and grown in culture. Reinjection of the cells in adult C57Bl/6 mice resulted in tumor induction 6–8 months later with a median survival of 170 days and with similar characteristics as the original tumor [22]. In order to try to reduce the survival time, tumor cells from a fully established invasive high-grade glioma (murine glioblastoma 0; mGB0) were isolated. Cells were serially implanted for two in vivo passages (mGB1 and mGB2) resulting in tumor development in all mice and a progressive shortening of the median survival. Based on genomic and transcriptomic data, mGB0 can be classified as the classical subtype, mGB1 as the proneural subtype and mGB2 as the mesenchymal subtype. mGB2 was selected as the most representative cell line compared to human disease with the worst prognosis and many histopathological features of high-grade gliomas. Also similar to what is observed in human GBMs, abundant myeloid cells and only few lymphocytes were found [179]. No therapies have been tested so far.

## 4. Conclusions

Immunocompetent mouse models are essential in preclinical GBM research, especially in the search for new immunotherapeutic strategies. When we compare all relevant mouse models based on their stemness, immune characteristics and response to standard-of-care treatment, it is clear that there is not one mouse model that perfectly recapitulates the heterogeneity of a human GBM tumor. However, the overview we present here can help in deciding which model is best suited for which type of research. For instance, the translational impact of research involving immune checkpoint blockade might not be recommended for a mouse model such as GL261 that is very sensitive to this type of immunotherapy (which is not compatible with the clinical situation). A better choice would be to use mouse models that show a certain degree of resistance to immune checkpoint blockade such as KR158B, 005 GSC, Nfpp10-GBM or SB28. In addition, if we want to take into account any type of model-intrinsic response to certain treatments, it would be even better to evaluate new treatment modalities in multiple models. Furthermore, different tumor models might correlate to different patient populations of GBM. Therefore, the heterogeneity of GBM would be better addressed in preclinical research if a heterogeneous composition of tumor models is used. Interestingly, a recent study highlighted a variable response to immune checkpoint inhibitors in syngeneic mice inoculated with the same type of cells (GL261) [180]. If individual factors are relevant in a standardize situation such as a syngeneic model, it is reasonable to expect that they play a dramatic role in actual patients. For all these reasons, we believe that understanding and modelling patients’ heterogeneity in preclinical research will be one of the most relevant challenges in future preclinical research for GBM.

Another outstanding question relates to how the genomic aberrations of each tumor model correlate to aberrations in its microenvironment, and, even more importantly, how this compares to the human situation. Indeed, ongoing trials for targeted therapies are mainly based on genomic matching; however, the identification of those patient populations with similar immunologic features as observed in the mouse models is still lacking, but could be key in targeting the right approaches to the right patients.

Lastly, it is striking that only very few preclinical studies have incorporated the standard-of-care regimen when testing new treatments. Moreover, in the limited cases where standard-of-care is taken into account, this usually only consisted of RT and/or TMZ and rarely included a surgical resection of the tumor, nevertheless the corner stone of the clinical treatment. It is well known that GBMs at first diagnosis and at recurrence (therefore, after the whole standard of care treatment) harbor important differences in term of molecular features and druggable targets [181]. In this view, it is of paramount importance to integrate in the pipeline of preclinical studies surgery, focal radiotherapy and TMZ-based chemotherapy in order to model such longitudinal neoplastic evolution. A paradigm shift is necessary: preclinical research should not only be aimed at discovering new treatments, but also at identifying the most appropriate momentum for their administration in order to maximize their effect in synergy with standard therapies.
